# Impact of depression on self-efficacy, illness perceptions and self-management among people with type 2 diabetes: A systematic review of longitudinal studies

**DOI:** 10.1371/journal.pone.0302635

**Published:** 2024-05-06

**Authors:** Andualem Derese, Yohannes Gebreegzhiabhere, Girmay Medhin, Sisay Sirgu, Charlotte Hanlon

**Affiliations:** 1 School of Public Health, College of Health and Medical Sciences, Haramaya University, Harar, Ethiopia; 2 Department of Psychiatry, School of Medicine, College of Health Sciences, Addis Ababa University, Addis Ababa, Ethiopia; 3 Department of Nursing, College of Health Sciences, Debre Berhan University, Debre Berhan, Ethiopia; 4 Aklilu Lemma Institute of Pathobiology, Addis Ababa University, Addis Ababa, Ethiopia; 5 Department of Internal Medicine, St. Paul’s Hospital Millennium Medical College, Addis Ababa, Ethiopia; 6 Health Service and Population Research Department and WHO Collaborating Centre for Mental Health Research and Training, Institute of Psychiatry, Psychology and Neuroscience, Centre for Global Mental Health, King’s College London, London, United Kingdom; 7 Centre for Innovative Drug Development and Therapeutic Trials for Africa (CDT-Africa), College of Health Sciences, Addis Ababa University, Addis Ababa, Ethiopia; Makerere University CHS: Makerere University College of Health Sciences, UGANDA

## Abstract

**Background:**

Treating comorbid depression does not always improve outcomes for people with type 2 diabetes. Evidence is lacking on potential psychological and behavioural intermediaries of the impact of depression on diabetes outcomes.

**Objective:**

To synthesise evidence on the impact of comorbid depression on self-efficacy, illness perceptions, and self-management in people with type 2 diabetes.

**Data sources:**

We searched PubMed, Embase, PsycINFO, and Global Health databases from inception up to 29^th^ March 2023.

**Study eligibility criteria:**

Only prospective studies (cohort or intervention studies) were included, with no restrictions on language. The outcomes were self-efficacy, illness perceptions, and self-management.

**Participants:**

People with type 2 diabetes in community or health settings.

**Exposure:**

Comorbid depression or depressive symptoms in people with type 2 diabetes.

**Synthesis of results:**

A narrative review of heterogeneous studies.

**Risk of bias:**

The risk of bias was assessed using the Effective Public Health Practice Project (EPHPP) quality assessment tool for quantitative studies.

**Results:**

Twenty-five studies were included, all from high-income countries. Depression was associated with lower self-efficacy (2 studies), poor illness perception (1 study), and poor self-management practices (17 studies) in people with type 2 diabetes. In 6/7 studies, depressive symptoms predicted less adherence to dietary recommendations, 8/10 studies found depressive symptoms were associated with poor medication adherence, 1/3 study found that depressive symptoms were associated with poor weight control, 3/4 with less physical exercise, and 2/3 with general self-care practices.

**Limitations:**

There were no studies from low- and middle-income countries and non-Western settings, and we cannot assume the mechanisms linking comorbid depression with diabetes outcomes are similar.

**Conclusions:**

Comorbid depression was associated with lower self-efficacy, poorer self-management, and less adaptive illness perceptions among people with diabetes.

## Introduction

Non-communicable diseases (NCDs) are among the leading causes of death globally, accounting for 71% of all deaths worldwide [1]. Diabetes mellitus (DM) is one of the five priority diseases targeted for action by world leaders at the United Nations General Assembly [2]. Diabetes is a chronic metabolic disorder characterised by persistent hyperglycemia [3]. Individuals with type 2 diabetes account for 90% of diabetes cases and have insulin resistance, usually accompanied by relative insulin deficiency [3]. Poorly controlled DM leads to severe complications, including stroke, blindness, kidney failure, and limb amputation [4].

Good glycemic control is crucial in avoiding diabetes complications. It can be achieved through practical diabetes self-management activities, such as regular plasma glucose monitoring, medication adherence, and diet and lifestyle modifications [5]. However, these self-care management activities are often complex, and many people with diabetes do not perform them as recommended [5,6].

People with diabetes are at a higher risk of mental health problems due to biological, environmental, social, behavioral, and emotional factors [7–9]. Depression often co-occurs with diabetes and has been found to adversely affect diabetes outcomes directly and indirectly [10–13]. A systematic review and meta-analysis of observational studies found that depression in people with diabetes mellitus ranged from 2% to 88%, with a pooled prevalence of 28% [14]. However, the nature of the relationship between depression and diabetes outcomes may be affected by intermediate factors [15–17], including psychosocial and behavioural factors such as self-efficacy [17,18], self-management [15,16,18], illness perception [19,20] and social support [21].

In systematic reviews and meta-analyses of comorbid depression and diabetes, interventions targeting depression have been found to reduce depressive symptom severity, but treating depression does not consistently improve glycemic control [22–25]. The reason might be that intermediate psychosocial and behavioural factors, specifically self-efficacy, illness perceptions, and self-care management, are negatively affected by depression but do not necessarily improve with interventions specific to depressive symptoms [15,18]. Systematic reviews have been published on the impact of comorbid depression on medication adherence in diabetes [26] and commitment to lifestyle changes [27], but not on the broader range of psychosocial and behavioural factors that may mediate the impact of depression on diabetes outcomes. Furthermore, these reviews are now outdated.

The objective of this review was to synthesise the evidence on the impact of comorbid depression on self-efficacy, illness perceptions, and self-management in people with diabetes.

## Materials and methods

### Registration and protocol

We followed the Preferred Reporting Items for Systematic Reviews and Meta-Analysis (PRISMA) guidelines [28] for reporting this review. The review protocol was registered on the PROSPERO International Register of Systematic Reviews [CRD42019136249].

### Search strategy

We searched PubMed, Embase, PsycINFO, and Global Health databases from inception to 29^th^ March 2023 without language restriction. We did forward and backward searches of the citations of included studies using Google Scholar and hand-searched for unpublished literature from University repositories. The search terms comprised key terms, MeSH terms, and Emtree terms for depression, diabetes mellitus, self-efficacy, illness perception, and self-management. The detailed search strategy is available in (S1 File).

### Eligibility criteria

The following criteria were used to include studies in the review:

**Study design:** Prospective cohort studies or intervention studies.

**Study setting:** Community, primary care, general and/or specialist medical outpatient settings. Studies conducted in in-patient settings were not eligible for this review.

**Participants:** Adults (age ≥ 18 years) with a clinical diagnosis (using HbA1C or plasma glucose) of type 2 diabetes mellitus. Studies that were restricted to special populations, including pregnant women, people with dementia or, psychosis, or HIV, were not eligible for this review.

**Exposure/intervention:** For observational studies, the exposure was the presence of depression assessed with either [A] diagnostic categories of depressive disorder based on the International Classification of Diseases (ICD-11 or earlier versions) or Diagnostic and Statistical Manual of Mental Disorders (DSM-V or earlier versions) criteria or [B] depressive symptoms identified using standardised scales like the Patient Health Questionnaire (PHQ-9) or Centre for epidemiologic studies depression scale (CES-D).

Intervention studies were included if they evaluated any intervention for participants with comorbid depression (diagnosed by a standardised tool or meeting diagnostic criteria). These interventions could be psychological, pharmacological, or a combination of both. For the intervention studies, our interest was in how changes in the exposure of depression (through intervention) affected the outcomes of self-efficacy, illness perceptions or self-management.

**Comparator:** People with a clinical diagnosis of type 2 diabetes mellitus without depression or elevated depressive symptoms.

**Outcomes:** The effect of depression on the constructs of self-efficacy, illness perceptions, or self-management, measured using standardised scales and defined as follows:

**Self-efficacy** is confidence in one’s ability to achieve intended results, according to Albert Bandura [29,30]. People differ in their efficacy across different domains of functioning. In the case of diabetes, self-efficacy is the judgment of one’s capabilities to monitor, plan, and perform self-management activities [31].

**Self-management** or self-care refers to daily activities that people with chronic diseases carry out to control their illnesses and cope with their illness’s psychosocial consequences [32]. Self-management comprises activities related to diet, exercise, blood glucose monitoring, medication adherence, and foot care [33].

**Illness perceptions** and **cognitions** are terms used to describe a range of cognitive processes underlying attention, interpretation, and behavior in response to illness-related information [34]. A positive perception of diabetes controllability, coupled with good knowledge about the disease, empowers individuals to adhere to treatment regimens and self-management practices like diet and exercise20].

### Screening

The identified references were first exported into Endnote reference management software [35], and duplicates were removed. The titles and abstracts were then independently screened for eligibility by two reviewers (AD and YG). The article was included for full-text review if screened in by either reviewer. Full manuscripts were checked independently against inclusion criteria by two authors, and any differences were reconciled through discussions. Excluded articles and reasons for exclusions were documented.

### Data extraction

Data were extracted by AD and YG independently into a custom-designed Excel spreadsheet with the following domains: author, publication year, country, study design, sample size, outcomes, measures, and critical findings. As before, any discrepancies were reconciled through discussions.

### Assessment of bias

Two of the co-authors (AD and YG) assessed the risk of bias independently using the Effective Public Health Practice Project (EPHPP) quality assessment tool for quantitative studies [36]. This tool has eight sections, of which six (selection bias, allocation bias, control of confounders, blinding of outcome assessors, data collection methods, and withdrawals and dropouts) were used in the global rating of the study. The global rating provides an overall methodological rating of strong, moderate, or weak [36]. While EPHPP dictionary provides detailed guidance for rating initial six components, it does not offer specific criteria for component ratings of intervention integrity and analysis appropriateness. For this study, we focused on evaluating each domain individually, to provide comprehensive understanding of the potential for bias. The issue was solved by discussion among the two authors (AD and YG) in case of discrepancies.

### Synthesis method

We used narrative synthesis. The included studies were critically appraised to assess their quality and relevance. Key findings were extracted from each study. Then, the studies were grouped together based on study design. The findings are then summarised in a narrative format, considering each study’s strengths and limitations.

## Results

### Study selection

**Fig 1:**
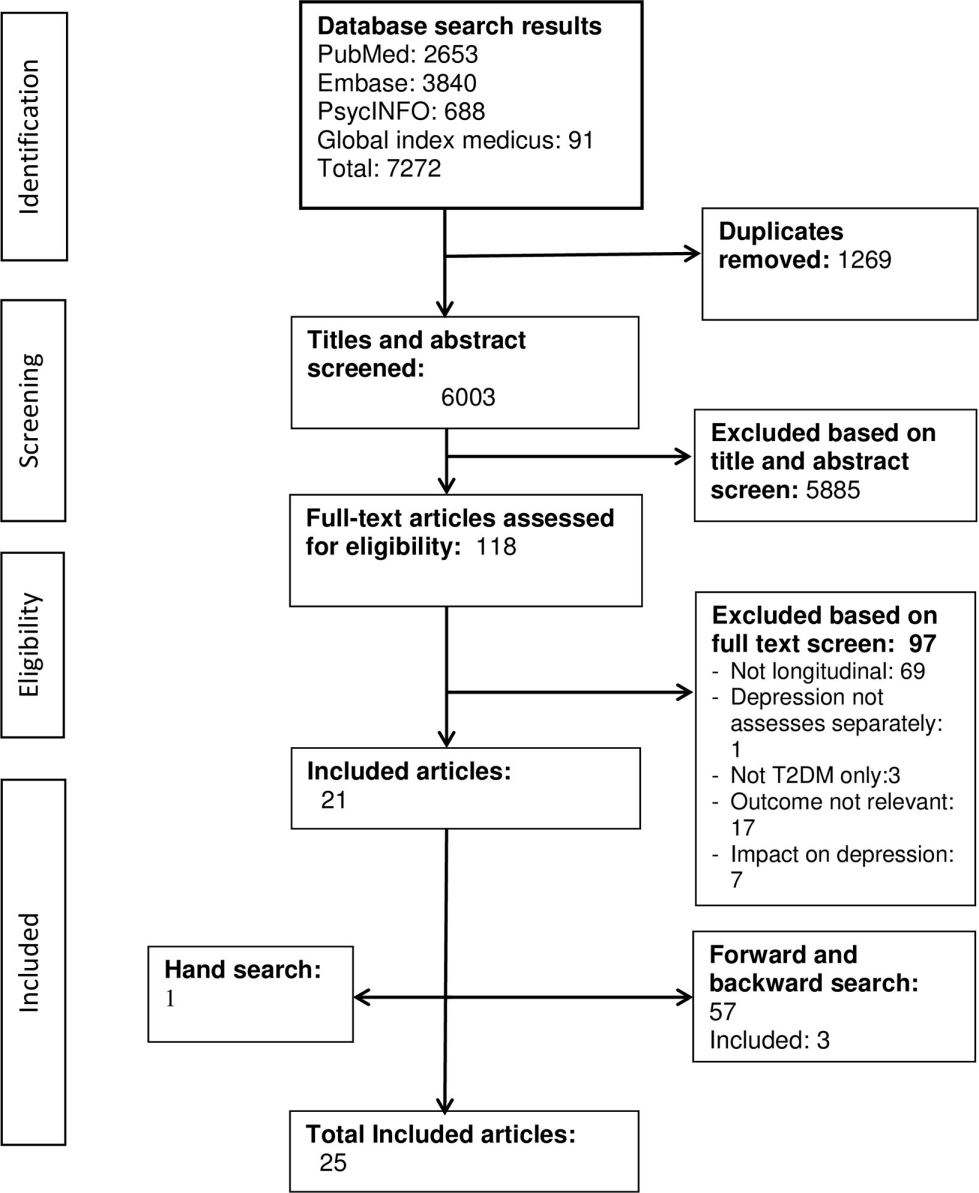
PRISMA flow diagram of the study selection process.

A total of 7272 records were obtained from four databases. After removing 1269 duplicates, 6003 records underwent title and abstract screening. Of these, 118 were included for full-text review, and 97 were excluded as they did not fulfill the inclusion criteria. Three further articles were identified from the forward and backward citation search, and one study was added through a hand search of unpublished databases. A total of 25 articles from 21 studies were included in the final analysis, as shown in the PRISMA diagram (Fig 1).

### Characteristics of the studies

All studies were from high-income countries (HIC) and were published from 2004 to 2022. Among the 25 included studies, the majority (17/25) were from the USA. The remaining studies were conducted in Canada (n = 4), Denmark, Germany, Japan, and the UK (one from each country).

Regarding study design, 18 were cohort studies, and seven were randomised controlled trials (RCTs). The sample size of each study ranged from 85 to 87,650. Most (n = 21) of the studies were facility-based, and only four were community-based.

The reviewed studies assessed the impact of comorbid depression on self-efficacy (n = 2), illness perceptions (n = 1), and different aspects of self-management (total n = 23), including medication adherence (n = 13), adherence to dietary recommendations (n = 11), exercise (n = 6), self-monitoring of blood glucose (SMBG)(n = 4), foot care (n = 3), weight change (n = 2), and compliance with regular visits (n = 1). (See Tables 1–3 below).

**Table 1:** Characteristics of the included studies with self-efficacy and illness perception as outcomes.

Author; Year; Country	Study design; sample size	Study setting	Population; sample size	Depression assessment tool	No. of people with depression	Treated for depression? (Y or N)	Outcome assessment tool	Outcomes
Oh H; 2018; USA [37]	Longitudinal analysis of an RCT; 251	Facility based	Adults with diabetes and probable depression; n = 251	PHQ-9	All	No	Self-Efficacy for Managing Chronic Disease scale	Changes in depressive symptoms predicted self-efficacy at the 6- and 12-month follow-ups.
Robertson SM; 2013; USA [38]	Longitudinal data from previous RCT; 85	Facility based	Patients with treated but uncontrolled T2DM	DASS	11 (10.3% of the total sample)	No	Diabetes Specific Self-Efficacy Scale	Depression was negatively related to Diabetes self-efficacy; stress was positively associated with Diabetes self-efficacy
Hudson JL; 2016; UK [39]	Longitudinal; 261	Facility based	Adults with T2DM under follow-up, n = 261	Diabetes Wellbeing Questionnaire (DWBQ)	NA	NA	revised Illness Perception Questionnaire (IPQ-R)	Depression and anxiety symptoms were associated with diabetes-related thoughts, but these thoughts did not affect diabetes management.

**Table 2:** Characteristics of the included RCTs with diabetes self-management behaviors as outcomes.

Author; Year; Country	sample size	Study setting	Population; sample size	Depression assessment tool	No. of people with depression	Treated for depression? (Y or N)	Outcome assessment tool	Outcomes
Hoogendoorn CJ; 2020; USA [40]	941	Population-based	Self-report of clinical diagnosis of T2DM; n = 941	PHQ-8^1^*	28% of the total sample	Yes (Telephone intervention)^2^*	Summary of Diabetes Self-Care Activities (SDSCA)	General psychological distress, rather than depression specifically, is related to diabetes self-care. The direct effect of depressive symptoms on diabetes self-care was not significant in the structural equation model (SEM).
Oh H; 2016; USA [41]	387	Facility based	Adults with DM and with MDD on OHA	PHQ-9 and HSC-20	All	Yes, ^3^*	SDCSA	Depression remission and receiving PST were not associated with a change in frequency in self-care behaviors in longitudinal analysis.
Oh H; 2014; USA [42]	387	Facility based	low-income, predominantly Hispanic adults with diabetes	PHQ-9	All	Yes (either EUC or collaborative depression care^4^*)	SDCSA	Decreased depression was associated with a more frequent healthy diet at the 12- (p < .01), 18- (p < .05), and 24-month follow-up (p < 0.05) and increased foot care at the 12- (p < .05) and 24-month follow-up (p < .01)
Wang ML; 2014; USA [43]	252	Facility based	Adult Latinos with a clinical diagnosis of T2DM	CES-D	45 mild; and 123 moderate-to-severe symptoms	Yes; ^5^*	items for diabetes self-management support	Study participants with moderate-to-severe depressive symptoms at baseline may have experienced the greatest improvement in dietary quality due to their decrease in depressive symptoms over time.
Lin EHB; 2006; USA [44]	329	Facility based	People with comorbid T2DM and depression	PHQ-9 for screening; SCL-90 for measuring change in depression	All (329)	Yes, ^6^*	SDSCA; computerised records of pharmacy refills (for medication adherence)	Enhanced depression care and outcomes were not associated with improved diabetes self-care behaviors (healthy nutrition, physical activity, or smoking cessation)
Williams Jr. JW; 2004; USA [45]	417	Facility based	Individuals over 60 years with diabetes and depression	SCID for diagnosis and SCL-20 for severity of depression	All (417)	Yes; ^7^*	SDSCA	-> 12 months of depression care improved depression-related outcomes and increased the frequency of exercise -> However, care management did not affect diet, diabetes medication adherence, glucose testing, or glycemic control
McKellar JD; 2004; USA [46]	307	Facility based	English/ Spanish speaking adults with T2DM	CES-D and 5-item mental health sub-scale from MOS-36	NA	NA	Morisky’s Medication Adherence Scale; 3 and 5 item measures developed by the authors for assessing diet and general self-care, respectively	-> An SEM model identified direct effects of baseline depressive symptoms on self-care and diabetes symptoms at follow-up ->The finding suggests that depressive symptoms have little direct impact on diabetes-related symptoms above and beyond their impact on patients self-care behaviors

^1^* The study used the 8-item version of the Patient Health Questionnaire, which excludes the question related to suicidal ideation.

2* Diabetes self-management support.

^3^* PST in collaborative depression care intervention on diabetes management.

^4^* The collaborative depression care was either anti-depressant medications or PST.

^5^* Group-based intervention guided by the social cognitive theory.

^6^* Collaborative depression care management (either anti-depressant medication or problem-solving treatment based on the patient’s choice).

^7^* Education, problem-solving therapy, or support for anti-depressant management; diabetes care was not specifically enhanced.

**Table 3:** Characteristics of the included observational studies with diabetes self-management behaviors as outcomes.

Author; Year; Country	Study design; sample size	Study setting	Population; sample size	Depression assessment tool	No. of people with depression	Treated for depression? (Y or N)	Outcome assessment tool	Outcomes
Brazeal M; 2022; USA [47]	Longitudinal; 625	Facility based	People with type 2 diabetes mellitus (T2DM); n = 625	PHQ-9	114	Yes (psycho-education and support)	SDSCA	No significant relationships were demonstrated between depression and any of the self-care activities.
Niaz D; 2022; Canada [48]	Longitudinal; 6201	Population-based	Adults with T2DM on metformin; n = 6201	Diagnostic codes	1621	Yes (Pharmacologic Treatment for Depression)	proportion of days covered (PDC) for measuring adherence to oral anti-hyperglycemic medications	After adjusting for other comorbidities and characteristics at baseline, people who started anti-depressant therapy were less likely to have poor adherence compared with controls (adjusted odds ratio, 0.85; 95% confidence interval [CI], 0.75 to 0.96; p = 0.007)
Rohde C; 2021; Denmark [49]	Longitudinal; 87650	Facility based	Adults with T2DM; n = 87650	Clinically diagnosed (ICD-10 or current use of anti-depressant)	784	Yes (Pharmacologic Treatment for Depression)	medication possession ratio (MPR) for adherence to medication	Compared with those without depression, depression or treatment with an anti-depressant was associated with an increased likelihood of glucose-lowering drug initiation and adherence, lipid-modifying agent initiation, and adherence.
Oh H; 2018; USA [37]	Longitudinal analysis of an RCT; 251	Facility based	Adults with diabetes and probable depression; n = 251	PHQ-9	All	NA	MOS Specific Adherence Recommendations (MOS-SAR) for adherence to self-care	Changes in depressive symptoms predicted level of adherence at the 6- and 12-month follow-ups
Lunghi C; 2017; Canada [50]	Longitudinal; 70633	Facility based	Adults with diabetes N = 73739	Clinically diagnosed a*	3106	NA	proportion of days covered (PDC) for medication adherence	Individuals with depression had a higher likelihood of nonadherence than those without depression [Adjusted Odds Ratio: 1.24 (95% Confidence Interval: 1.13–1.37)]
Gentil L; 2016; Canada [51]	Longitudinal; 301	Community-based	Adults with T2DM, aged 65 and over, taking oral hypoglycemic agent (OHA) N = 301	ESA ^b^*	NA	NA	medication possession ratio (MPR) for medication adherence	A latent growth curve model showed no significant difference in medication adherence to oral hypoglycaemic agents between participants with and without depression or anxiety disorders.
Messier L; 2013; Canada [52]	Longitudinal; 387	Community-based (Telephone interviews)	Adults with T2DM on OHA	PHQ-9	136	NA	Author developed questions for exercise, diet and body weight control	Individuals who developed depression were found to be more likely to be inactive at the beginning of the study, remain inactive at the one-year follow-up, and report a worsening perception of controlling body weight. ->Additionally, they reported maintaining a poor perception of controlling the amount of food eaten and their body weight compared to those who did not develop depression (p<0.05).
Hernandez R; 2013; USA [53]	Longitudinal; 276	Facility based	Latino and African American adults with T2DM	PHQ-9	62	No	SDSCA	After adjusting for the baseline value: -> Baseline depression associated with fewer days per week of the behavior associated with general diet (i.e., following a healthful eating plan) at 6 and 18 months PR/Mean difference = 0.67 & 0.68, respectively [but no difference at 12 months) ->There is a significant difference in following specific diet recommendations (i.e., fruit and vegetable consumption and decreased high-fat food consumption) at 12 months, with PR/Mean difference = 0.7. ->When depression is treated as a continuous variable, depression was only significantly associated with the outcome measure of specific diet (B = 0.0007) ->In multivariate analysis, changes in depression level were only associated with changes in specific diet and physical exercise (B = -0.037 & -0.070 respectively)
Dirmaier J; 2010; Germany [54]	Longitudinal; 866	Facility based	Adults with T2DM	The Depression Screening Questionnaire (DSQ)	Subthreshold depression = 179,Depression = 102	There is no data on the course or treatment of depression during the 12-month follow-up period	Author developed measures for medication adherence and non-adherence for health behaviour	->Subthreshold depression at baseline was not associated with medication adherence at 12 months ->Having depression at baseline increased the odds of having problems with medication adherence at 12 months [(OR = 2.67; CI: 1.38–5.15; p = 0.003)] ->After adjusting for covariates, subthreshold depression, and depression at baseline predicted increased problems with diabetes-related health behaviors at 12 months (b = 1.01; CI: 0.62–1.40) and (b = 0.96; CI: 0.40–1.52; R 2 = 0.30) respectively.
Chiu Ching-JU; 2010; USA [55]	Longitudinal; 998	Facility-based + mail based	middle-aged and older people with Diabetes (aged 51 and above at baseline)	CES-D and CIDI short form (CIDI-SF)	NA	NA	Author developed measures for exercise, body weight control, and current smoking status	->No change in health behavior index for those with no/low depression, but the score decreased significantly for those with moderate and high depression at 2nd year follow up ->On mediation analysis, the indirect effect of depressive symptoms on HbA1c levels through health behaviors was significant (b indirect = -0.09 9–0.17 = 0.015) ->Health behaviors accounted for 13% of the association between depressive symptoms and HbA1c levels (b indirect/b total = 0.015/0.115 = 0.13)
Katon W; 2010; USA [56]	Longitudinal; 2759	Facility based	Patients with filled prescriptions for insulin or OHA	PHQ-9	382	No	SDSCA	Individuals with persistent or worsening depressive symptoms over five years demonstrated either continued difficulty adhering to diet and exercise regimens or a decline in adherence over time, compared to those with low levels of depressive symptoms
Hayashino Y; 2010; Japan [57]	Longitudinal data from an RCT; 1444	Facility based	Adults with T2DM	CES-D	133	No	Questions derived from Diabetes Quality Improvement Project (DQIP) for adherence with appointment keeping for diabetes care	The multivariable-adjusted hazard ratio of poor compliance with regular visits in those having depressive symptoms was insignificant (hazard ratio: 1.23, 95% CI: 0.46–3.31) -> However, there was a statistically significant higher risk of poor compliance in those not completing the questionnaire (hazard ratio: 2.26, 95% CI: 1.94–2.63)
Ludman EJ; 2009; USA [58]	Longitudinal; 2600	Facility based	Adults with T2DM	PHQ-9	Baseline PHQ ≥ 10 (n = 525)	Only some of them were taking treatment for depression	chronic disease score (RxRisk)^C^*	->There was no significant difference in weight change patterns between individuals who saw a decline in their depressive symptoms and those who consistently had low depression symptoms. -> Individuals who had an increase in depression symptoms did not exhibit a different pattern of weight change compared to those who consistently had low levels of depression symptoms.
Katon W; 2009; USA [59]	Longitudinal; 4117	Facility based	Patients with filled prescriptions for insulin or OHA	PHQ-9	495	NA	chronic disease score (RxRisk) ^C^*	For people with diabetes and poor disease control, the presence of depression was associated with a higher probability of: ->Poor adherence to diabetes control medications (OR [95% CI] = 1.98 [1.31, 2.98]), Antihypertensives (OR [95% CI] = 2.06 [1.47,2.88]), and LDL control medications (OR [95% CI] = 2.43 [1.19, 4.97])
Gonzalez JS; 2008; USA [60]	Longitudinal;208	Facility based	Adults with T2DM	HANDS	30 at baseline and 81% of the baseline at the follow-up	No	SDSCA	Higher baseline depressive symptoms significantly predicted: ->Worse adherence to general dietary recommendations, medication nonadherence, less spacing of carbohydrates, lower consumption of fruits and vegetables, less exercise, less frequent SMBG, and worse foot care at follow-up ->This association persisted even after controlling for baseline levels of self-care
Kilbourne AM; 2005; USA [61]	Longitudinal; 203	Facility based	People with T2DM on OHA	PHQ-9	19 (10%)	NA	prescription refill data and brief adherence question	After adjusting for demographic and substance use variables: ->Depression was associated with poor adherence based on patient reports (OR = 0.2; P = 0.02). ->Depression was associated with worse adherence, based on pharmacy data (b = –20; p = 0.04) (i.e., those with depression showed 20% fewer days with adequate medication coverage) ->Depression was not significantly associated with adherence based on provider reports or EMC data after adjustment

a* Taken from the chart through ICD-9 codes or evidence of anti-depressants.

^b^* ESA-Questionnaire (ESA-Q)—a tool similar to CIDI that uses DSM-IV criteria.

c* Rx Risk—Chronic disease score derived from pharmacy records. This score reflects medical comorbidity based on medications prescribed in the past year.

OHA- Oral hypoglycaemic agents; MDD- Major depressive disorder; PHQ-9 –Patient health questionnaire; HSC-20 –Hopkins symptom checklist; CIDI–Composite international diagnostic interview; CES-D–Centre for epidemiologic studies depression scale; SCL-90—Hopkins Symptom Checklist 90; SCID–Structured clinical interview; DASS—The Depression, Anxiety and Stress Scale; NA–information not available.

Seven screening tools and four approaches to diagnostic measurement were used to define depression. The screening scales used were the Patient Health Questionnaire (PHQ-9), Center for Epidemiologic Studies Depression Scale (CES-D), Depression, Anxiety and Stress Scale (DASS), Diabetes Wellbeing Questionnaire (DWBQ), ESA-Questionnaire (ESA-Q), Depression Screening Questionnaire (DSQ), Hopkins Symptom Checklist 90 (HSC-90), Harvard Department of Psychiatry/National Depression Screening Day Scale (HANDS) and 5-item mental health sub-scale from Medical Outcomes Study (MOS-36 items). The diagnostic instruments for depression were the Composite International Diagnostic Interview (CIDI), Emotional Self-Awareness Questionnaire, and Structured Clinical Interview for DSM (SCID) or trained clinicians applying international diagnostic criteria.

The studies used diverse measures, methods, and analytic approaches, meaning a meta-analysis could not be carried out.

### Risk of bias within studies

Most studies received “strong” ratings for data collection methods and handling of confounders. Withdrawals and dropouts led to weak ratings and a risk of attrition bias. (See S2 File).

### Effect of depression on self-efficacy

Two studies reported the effect of depression on self-efficacy [37,38]. Both studies were from the USA and were longitudinal analyses nested within a trial. The studies reported either change in depressive symptoms or baseline depression being negatively and significantly associated with self-efficacy at follow-up after adjusting for relevant covariates.

### Effect of depression on illness perception

Only one cohort study reported the impact of depression on illness perceptions after six months of follow-up. In the study by Hudson et al. [39], depression and anxiety symptoms at baseline were prospectively associated with specific diabetes illness perceptions. Participants with higher depression scores and who were more anxious at baseline were more likely to perceive that diabetes was an unpredictable condition at six months follow-up [39].

### Effect of depression on diabetes self-management

Twenty-two articles assessed the effect of depression on adherence to medication, dietary recommendations, physical exercise, health behaviors, glucose testing, weight loss, foot care, and reducing substance use. The findings are described as follows:

### Effect of depression on adherence to medication

Ten studies (eight cohort studies and two RCTs) assessed the effect of depression on medication adherence. Baseline depressive symptoms were significant predictors of poor adherence to diabetes control medications [44,50,59–61], anti-hypertensive medications [59], and low-density lipoproteins (LDL) control medications [59]. Another study found that having depressive symptoms at baseline predicted problems with medication adherence at consecutive follow-ups [54]. A study in Denmark found that the presence of depression or treatment with an anti-depressant was associated with an increase in diabetes medication initiation and adherence when compared to those without depression treatment [49]. Another study also found that people who started anti-depressant therapy were less likely to have poor adherence compared with controls who did not take anti-depressants [48].

On the contrary, two studies found no longitudinal association between depressive symptoms and adherence to oral hypoglycemic agents [45,51]. In a randomised, controlled trial that assessed the effectiveness of depression care management to improve affective and diabetes outcomes in older adults, enhanced care was associated with reduced severity of depression but no impact on medication adherence [45].

### Effect of depression on dietary recommendations

In six studies, baseline depressive symptoms significantly predicted poor adherence to general dietary recommendations [42,52–54,56,60], decreased spacing of carbohydrates [53,60], and lower consumption of fruits and vegetables [53,60] at follow-up, after controlling for baseline levels of self-care. Similarly, participants with moderate-to-severe depressive symptoms at baseline had significant improvements in dietary quality when their depressive symptoms decreased over time [43]. In contrast, depression care interventions in three studies did not affect adherence to dietary recommendations [44,45,47].

### Effect of depression on physical exercise

In six studies, depressive symptoms were significantly associated with less physical exercise or weight loss [52–54,56,58,60]. In another study, depression care interventions increased the frequency of exercise [45], but a 12-month enhanced depression care intervention was not associated with improvement in physical activity [44].

### Effect on substance use

In a cohort study, depression and sub-threshold depression at baseline significantly predicted increased use of substances, like cigarette smoking and alcohol consumption, at follow-up [54]. Lin et al. assessed the impact of a 12-month enhanced depression care intervention, which was not associated with improvement in smoking cessation [44].

### Effect on health behaviors

In eight studies, depression longitudinally predicted less frequent self-monitoring of blood glucose [60], poorer compliance with regular visits [57], and worse foot care [42,60,62] at follow-up, controlling for baseline levels of self-care. In three studies, baseline depressive symptoms had a direct negative effect on overall self-care at follow-up [46,54,55]. In an RCT, a physical exercise intervention for depression significantly improved diabetes distress, diabetes self-care, and quality of life in individuals with diabetes. Additionally, the program led to reductions in triglycerides, total cholesterol and LDL-cholesterol [63]. However, depression care interventions in another trial did not affect glucose testing or glycemic control [45]. Similarly, one study demonstrated no significant relationship between depression and self-care activities [47].

Problem-solving therapy (PST) for depression was not associated with a change in the frequency of self-care behaviors [41]. PST intervention and depression remission (assessed using PHQ-9) from the baseline did not result in a significant change in the frequency of self-care behaviors in two prospective models (from baseline to 18 months and from baseline to 24 months) [41]. However, depression remission status assessed by SCL-20 significantly predicted a more regular healthy diet and exercise at follow-up [41].

## Discussion

In this systematic review, we synthesised findings from studies on the impact of comorbid depression in people with type 2 diabetes mellitus on self-efficacy, illness perceptions, and self-management. All included studies were longitudinal, with either interventional or observational cohort designs, and were of good quality; however, none were conducted in LMICs. Comorbid depression in people with type 2 diabetes mellitus (T2DM) predicted low self-efficacy, pessimistic illness perception, poorer adherence to medication, and greater difficulty in following dietary recommendations in most, but not all, studies. Interventions for depression resulted in improvement in depressive symptoms. However, improvement in depressive symptoms did not consistently lead to improvements in DM self-management.

The two studies that assessed the impact of depression on diabetes self-efficacy indicated that depression at baseline was negatively associated with self-efficacy at follow-up. This is in keeping with the predictions of self-efficacy theory and highlights that emotional states can boost or undermine self-efficacy [64]. A reciprocal relationship between depression and specific diabetes cognitions over time, with those who were depressed more likely to perceive DM as unpredictable [39], is consistent with the Common Sense Self-Regulation Model (CS-SRM) [65]. This could be because a higher level of depression is associated with lower perceived personal and treatment control and more severe consequences of chronic disease [66]. However, a study from the UK that used structural equation modeling to investigate potential mechanisms found that depression was directly associated with specific diabetes cognitions over time but that these cognition domains did not mediate the effect of emotion on diabetes self-care [39]. Further prospective studies are needed to investigate how self-efficacy relates to depression and self-management activities.

The review findings indicated that depressive symptoms generally predicted lower engagement with self-management behaviors, but the evidence was stronger for some types of self-management behaviors than others. For example, depressive symptoms were consistently associated with lower adherence to medication, exercise and diet, compared to the association with weight control. This is in line with Beck’s cognitive model, which hypothesises that people’s emotions and behaviors are influenced by their perceptions of events [67]. According to the cognitive model, individuals’ perceptions are often distorted and dysfunctional when distressed or depressed, affecting their physiological and behavioral actions, including motivation to carry out self-care activities [67].

However, the review findings indicate that not all interventions targeting depression improved self-management behaviors. The pathway through which depression affects diabetes outcomes may not only be directly through its effect on self-management; other indirect mechanisms might also be involved through diabetes distress or self-efficacy [68]. The other explanation might be that the intensity of these interventions might not be sufficient to increase optimism sustainably and the DM-specific motivation required to bring change related to self-management behaviors, even if depressive symptoms have reduced.

There were different interventions targeting either depression alone or both depression and diabetes management. In an RCT by Lin et al., a collaborative depression intervention (pharmacotherapy, problem-solving treatment, or combination) was used. The intervention resulted in less severe depression over time but did not result in improved diabetes self-care behaviors when compared to the usual care [44]. On the contrary, Oh et al. found that Problem Solving Therapy (PST) for depression did not result in depression remission. Furthermore, depression remission and receiving PST were not associated with changes in self-care behaviors [41]. Another study using a diabetes self-management intervention resulted in a lower mean CES-D score over time than the usual care, indicating potential bi-directional association [43].

Williams et al. used enhanced care (that included education, PST, or support for anti-depressant management) for depression. They found that the intervention resulted in less severe depression and improved overall functioning compared to the usual care. Moreover, the intervention increased weekly exercise days but did not affect other aspects of self-care [45].

In this review, we focused on individual psychological constructs, which may be less salient in other sociocultural settings, particularly those more collectivistic. In such settings, self-management may rely more on others and available resources than just the individual, as seen in managing other chronic conditions [69]. General illness perceptions about diabetes mellitus may be more externalised in some non-Western settings (i.e., less in the control of the individual). Therefore, the interaction with depressive symptoms may have different consequences. Nonetheless, a qualitative study in Ethiopia among people with type 2 DM indicated that an individual’s perception of their illness and its treatment could negatively influence their experience and adherence to medication [70]. Further work is needed to understand salient contextual factors for people with DM and comorbid depression in LMICs and the potential mechanisms linking these constructs to DM outcomes.

## Limitations

Our review identified no studies from LMICs or non-Western countries. There is, therefore, a critical gap in the research evidence. LMIC and non-Western settings are likely to differ in terms of social, cultural, and educational factors, which could influence the interplay between depression, self-efficacy, illness perceptions, and self-management. There were methodological differences in the included studies, which precluded meta-analysis and limited the conclusions that could be drawn from the review. There were very few studies on the impact of depression on illness perception and self-efficacy. The potential for selection bias, specifically attrition bias, also limited the review, as only studies published in English were included. This could have excluded studies that were published in other languages.

## Conclusions

Observational studies generally suggest that depressive symptoms are associated with lower self-efficacy and engagement in self-management behaviours, but the evidence is mixed. Interventions designed to reduce depressive symptoms have also shown mixed results regarding their impact on self-management behaviours. Further research is needed to identify the pathways through which depression affects diabetes outcomes to inform intervention development. There is a particular need for studies from LMICs and non-Western settings where mechanisms linking comorbid depression with diabetes outcomes may differ.

## Supporting information

S1 File Search strategy used in the systematic review. (DOCX)

S2 File Quality assessment for included studies. (DOCX)
