# Supplementary material for: Impact of depression on self-efficacy, illness perceptions and self-management among people with type 2 diabetes: A systematic review of longitudinal studies
Source: PLoS One. 2024 May 6;19(5):e0302635. doi: 10.1371/journal.pone.0302635 (PMC11073729; doi:10.1371/journal.pone.0302635)
Supplement: S1 File — (DOCX) [file pone.0302635.s001.docx]

Supporting file 1: Search strategy for systematic review of impact of depression on self-care, self-efficacy, illness perceptions and diabetes outcomes in people with type 2 diabetes

| Big terms |  | |
| --- | --- | --- |
| **Big term 1:**  Depression | Free terms | Depres* OR “Major depressive disorder” OR "depressive symptom" OR "depressive state" OR "depressive disorder" OR "depression disorder" OR distress OR “mental health” |
|  | MeSH terms | "Depression"[Mesh] OR "Depressive Disorder"[Mesh] OR "Depressive Disorder, Major"[Mesh] OR “Mental health”[Mesh] |
|  | Free term (Embase) | Depress* or dysthym* or mood disorder or mental health |
|  | Emtree terms | exp major depression/ or exp "mixed anxiety and depression"/ or exp depression/ or exp minor depression/ or exp bipolar depression/ |
|  | Psych-INFO terms | Depression or dysthymi$ or (depressi$ adj3 disorder$) or (depressi$ adj3 symptom$) |
| **Big term 2:**  Diabetes | Free terms | “Type 2 diabetes” OR “type II diabetes” OR “Diabet* AND type 2”OR “Diabet* AND type II” OR "type 2 DM" OR T2DM OR “noninsulin-dependent diabetes mellitus" OR niddm OR t2d |
|  | MeSH terms | "Diabetes Mellitus"[Mesh] OR "Diabetes Complications"[Mesh] OR "Diabetes Mellitus, Type 2"[Mesh] |
|  | Free term (Embase) | non insulin dependent diabetes mellitus OR (Type* adj3 ("2" or "II" or two*) adj3 (diabete* or diabetic*)) OR (adult* onset* adj3 (diabete* or diabetic*)) OR ((Ketosis-resistant* or stable*) adj3 (diabete* or diabetic*)) OR ((Non-insulin* or Non insulin* or Noninsulin*) adj3 depend* adj3 (diabete* or diabetic*)) OR NIDDM.tw OR T2D |
|  | Emtree terms | exp diabetes mellitus/ |
|  | Psych-INFO terms | TYPE 2 DIABETES/ or (Type* adj3 ("2" or "II" or two*) adj3 (diabete* or diabetic*)) or (adult* onset* adj3 (diabete* or diabetic*)) or ((Ketosis-resistant* or stable*) adj3 (diabete* or diabetic*)) or ((Non-insulin* or Non insulin* or Noninsulin*) adj3 depend* adj3 (diabete* or diabetic*)) or NIDDM or t2d |
| **Big term 3:**  Self-care OR Self-efficacy OR Illness perception | Free terms | Self*care OR self*manag* OR self*Monitor OR “self management” OR “self-management” OR adherence OR compliance OR “self efficacy” OR “self-efficacy” OR “self concept” OR self-concept OR “illness perception” |
|  | MeSH terms | "Self Care"[Mesh] OR "Self-Management"[Mesh] OR "Self Efficacy"[Mesh] |
|  | Free term (Embase) | self care or (self-care or self-management or self-monitor* or self-help) or self efficacy or illness perception |
|  | Emtree terms | exp self care/ or exp self concept/ or illness perceptions.mp. |
|  | Psych-INFO terms | self care or (self-care or self-management or self-monitor* or self-help) or self efficacy or illness perception or exp Self-Management/ or exp Self-Care Skills/ or exp Diabetes Mellitus/ or exp Health Behavior/ |
| **Final Search** | Big term 1 AND Big term 2 AND Big term 3 | |

***Abbreviation:*** MeSH, medical Subject Headings in MEDLINE
