# Supplementary material for: Impact of depression on self-efficacy, illness perceptions and self-management among people with type 2 diabetes: A systematic review of longitudinal studies
Source: PLoS One. 2024 May 6;19(5):e0302635. doi: 10.1371/journal.pone.0302635 (PMC11073729; doi:10.1371/journal.pone.0302635)
Supplement: S2 File — (DOCX) [file pone.0302635.s002.docx]

Supporting File 2: Quality assessment for Impact of depression on self-efficacy, illness perceptions and self-management among people with type 2 diabetes: a systematic review

| S.N. | **Author (Year)** | **Criteria** | | | | | |
| --- | --- | --- | --- | --- | --- | --- | --- |
|  |  | **Selection bias** | **Study design** | **Confounders** | **Blinding** | **Data Collection method** | **Withdrawals and dropouts** |
| 1 | Chiu CJ; 2010 | 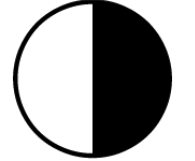 | 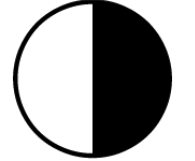 | 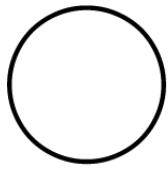 | 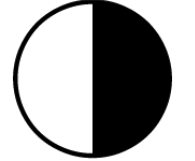 | 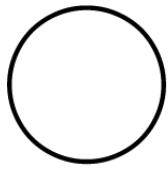 | 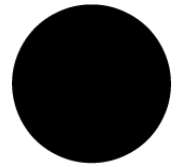 |
| 2 | Gentil L; 2016 | 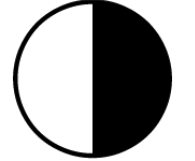 | 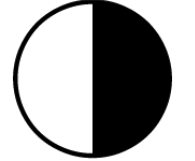 | 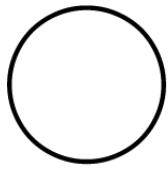 | 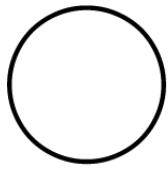 | 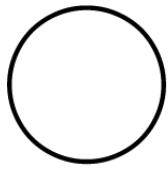 | 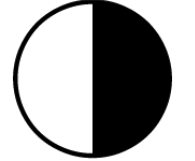 |
| 3 | Gonzalez JS; 2008 | 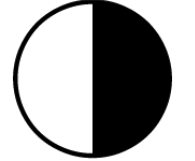 | 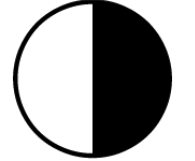 | 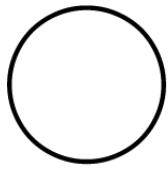 | 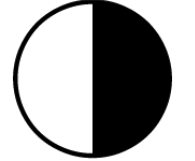 | 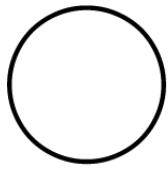 | 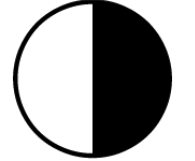 |
| 4 | Hayashino Y; 2011 | 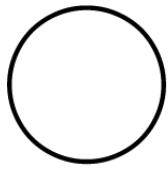 | 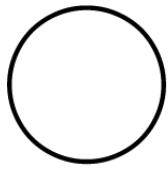 | 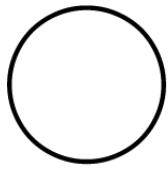 | 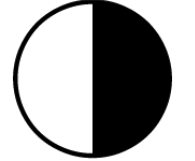 | 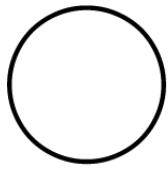 | 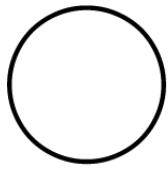 |
| 5 | Hernandez R; 2013 | 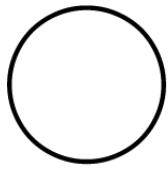 | 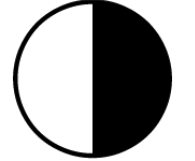 | 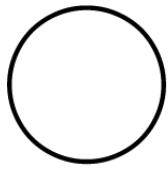 | 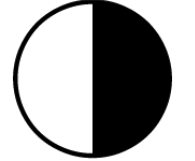 | 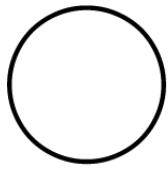 | 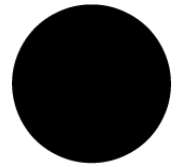 |
| 6 | Katon W; 2009 | 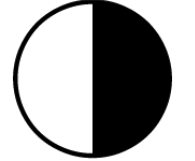 | 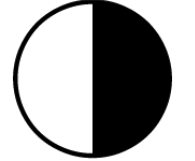 | 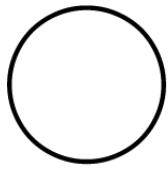 | 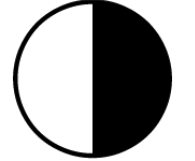 | 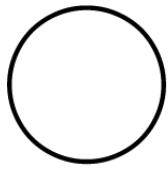 | 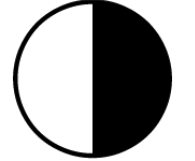 |
| 7 | Katon W; 2010 | 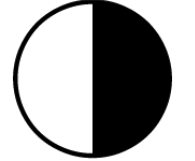 | 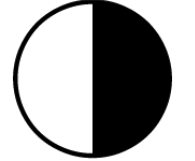 | 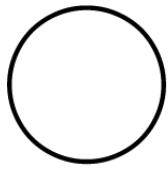 | 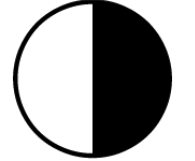 | 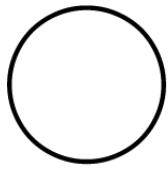 | 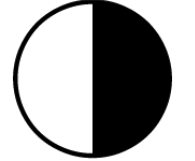 |
| 8 | Hudson JL; 2016 | 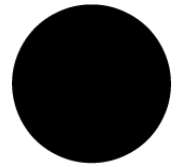 | 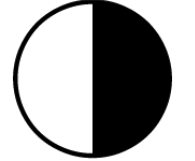 | 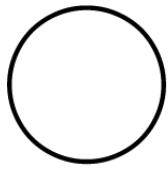 | 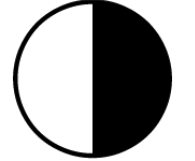 | 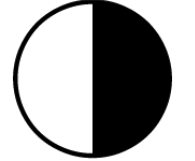 | 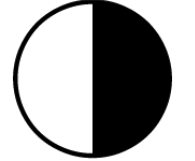 |
| 9 | Kilbourne AM; 2005 | 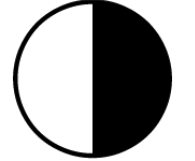 | 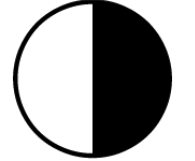 | 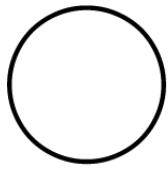 | 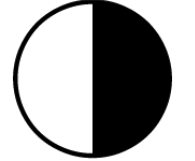 | 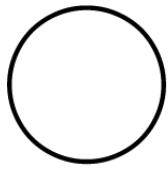 | 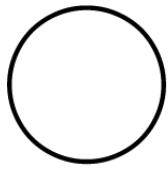 |
| 10 | Lin EHB; 2006 | 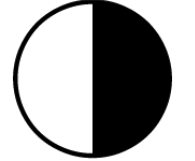 | 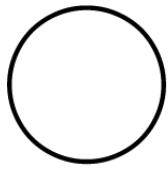 | 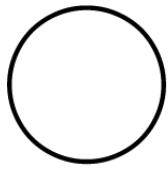 | 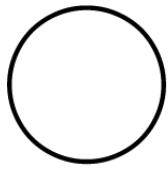 | 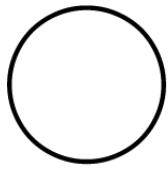 | 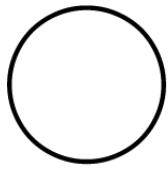 |
| 11 | Lunghi C; 2017 | 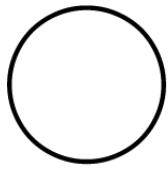 | 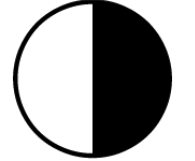 | 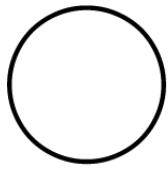 | 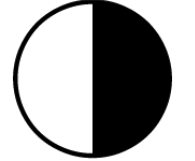 | 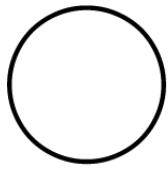 | 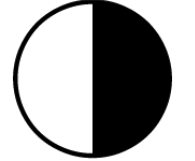 |
| 12 | McKellar JD; 2004 | 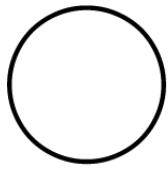 | 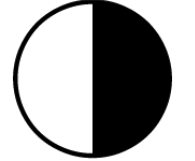 | 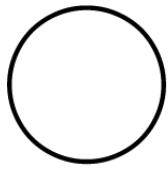 | 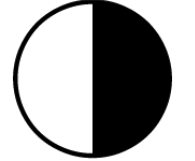 | 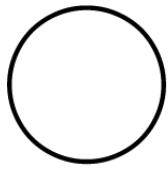 | 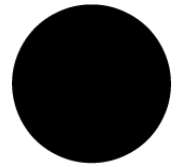 |
| 13 | Oh H; 2016 | 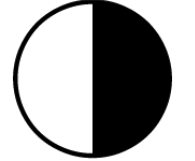 | 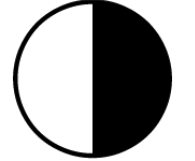 | 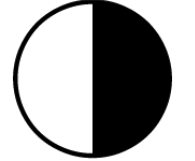 | 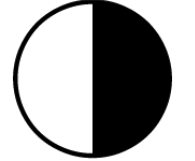 | 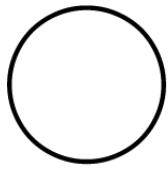 | 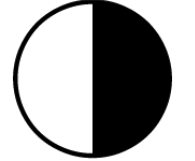 |
| 14 | Oh H; 2018 | 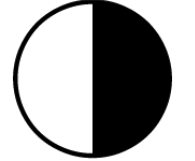 | 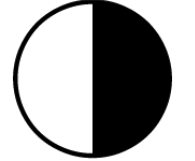 | 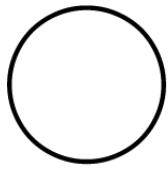 | 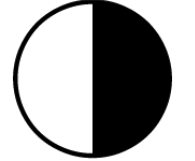 | 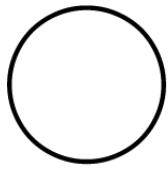 | 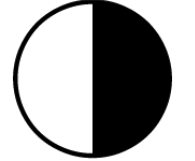 |
| 15 | Robertson SM; 2013 | 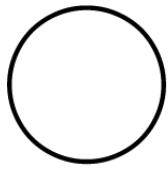 | 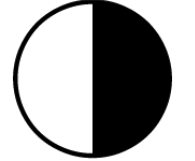 | 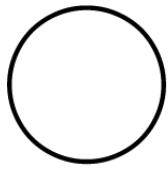 | 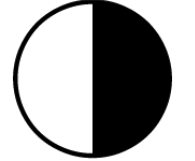 | 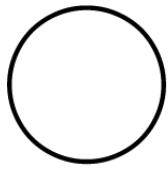 | 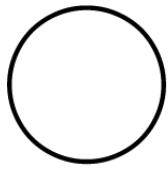 |
| 16 | Wang ML; 2014 | 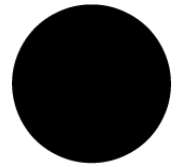 | 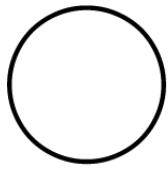 | 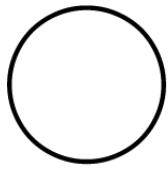 | 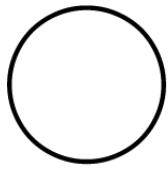 | 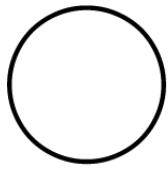 | 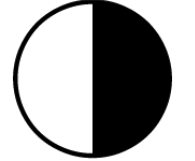 |
| 17 | Williams JW; 2004 | 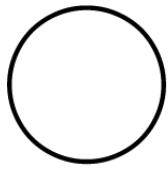 | 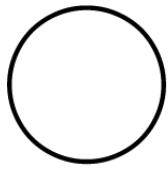 | 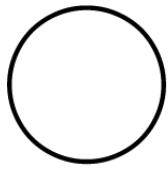 | 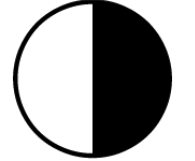 |  |  |
| 18 | Dirmaier J; 2010 |  |  |  |  |  |  |
| 19 | Ludman EJ; 2009 |  |  |  |  |  |  |
| 20 | Messier L; 2013 |  |  |  |  |  |  |
| 21 | Oh H; 2014 |  |  |  |  |  |  |
| 22 | Hoogendoorn CJ; 2020 |  |  |  |  |  |  |
| 23 | Rohde C; 2021 |  |  |  |  |  |  |
| 24 | Brazeal M; 2022 |  |  |  |  |  | NA |
| 25 | Niaz D; 2022 |  |  |  |  |  | NA |

= Strong

= Moderate

= Weak

NA= Not Applicable
